# Bone health in young adults with type 1 diabetes and progressive eGFR decline

**DOI:** 10.1186/s40842-024-00169-6

**Published:** 2024-05-25

**Authors:** Funmbi Babalola, Jill Hamilton, Michael Zappitelli, Yesmino Elia, Jacqueline Curtis, Rahim Moineddin, Farid H. Mahmud

**Affiliations:** 1 Division of Endocrinology, Department of Pediatrics, The Hospital for Sick Children, University of Toronto, Toronto, ON Canada; 2 SickKids Research Institute, Temerty Faculty of Medicine, University of Toronto, Toronto, Canada; 3 Division of Nephrology, Department of Pediatrics, The Hospital for Sick Children, University of Toronto, Toronto, ON Canada; 4 Department of Family and Community Medicine, University of Toronto, Toronto, ON Canada

**Keywords:** Diabetes mellitus, Type 1, Tomography scanners, X-ray computed, Bone mineral density, Bone fractures, Diabetic kidney disease

## Abstract

**Background:**

Type 1 Diabetes (T1D) is associated with increased risk of fractures, worsened by presence of microvascular complications. This study’s objective is to determine the impact of progressive decline in estimated glomerular filtration rate (eGFR) on bone biomarkers and bone microarchitecture in youth with T1D.

**Methods:**

Slopes of eGFR were calculated using measures obtained at four timepoints from adolescence to young adulthood. Participants were identified as eGFR decliners if eGFR decreased ≥ 3ml/min/1.73m^2^/year. Bone health was assessed in young adulthood by high resolution peripheral quantitative computed tomography (HRpQCT Xtreme CTII) and bone biomarkers; osteocalcin, procollagen 1 intact n-terminal pro-peptide (P1NP), c-terminal telopeptide (CTX), and bone specific alkaline phosphatase. The relationship between diabetes duration, glycated hemoglobin, body mass index (BMI) and vitamin D level on bone biomarkers and microarchitecture was evaluated. Linear regression analysis was used for the statistical analysis in this study.

**Results:**

Ninety-nine study participants were studied with longitudinal evaluation of eGFR over 7.4 ± 1.0 years with mean age of 14.7 ± 1.7 years at baseline. Cross sectional evaluation of bone was performed at 21.3 ± 2.1 years. 44% participants had eGFR decline and showed 5% higher cortical porosity diameter than non-decliners (*p* = 0.035). Greater diabetes duration was associated with higher trabecular separation (*p* = 0.004) and lower trabecular number (*p* = 0.01). Higher level of 25 hydroxy-vitamin D was associated with lower trabecular separation (*p* = 0.01). Elevated glycated hemoglobin (*p* = 0.0008) and BMI (*p* = 0.009), were associated with lower markers of bone formation.

**Conclusion:**

Mild increase in cortical porosity diameter was found in youth with T1D and eGFR decline, however, overall measures of bone microarchitecture on HR-pQCT were similar between both groups and there were no statistically significant changes in bone biomarkers. Hence, skeletal impairments were limited in youth with different eGFR trajectories near peak bone mass. Longitudinal HR-pQCT studies are needed to further understand the impact of eGFR decline on bone microarchitecture. Optimal glycemic control, normal BMI and vitamin D status were supported by this study as important markers for good bone health.

## Background

Type 1 diabetes (T1D) is associated with increased risk of fractures that is 2 to fourfold higher relative to the general population [1]. Fracture risk is increased in the presence of diabetic kidney disease (DKD) [2]. Newer imaging modalities, such as high resolution peripheral computed tomography (HRpQCT) have improved sensitivity compared to commonly used imaging modalities to evaluate and detect changes in bone microarchitecture [3]. HRpQCT is a non-invasive imaging modality that provides information on bone mineral density with additional information on trabecular and cortical bone compartments [3]. HRpQCT studies in adolescents with T1D have shown significant differences compared to healthy controls including lower tibia trabecular thickness and higher tibia cortical porosity consistent with reduced bone quality [4]. Furthermore, HRpQCT studies in adults report markers of worsened bone microarchitecture in adults with T1D compared to healthy controls [2]. Additional impairments, including lower total volumetric bone mineral density, trabecular volumetric bone mineral density, trabecular thickness and failure load and increased trabecular separation, have been shown in T1D patients with microvascular complications compared to those without microvascular complications [2].

Albuminuria is a well-recognized and clinically evaluated marker to assess diabetes-related kidney changes. However, there is evidence of changes in kidney function of patients with T1D prior to development of microalbuminuria or eGFR < 60ml/min/1.73m^2^ [5–7]. Progressive decline in eGFR is reported to be a predictor of progression to DKD that can be used as a surrogate marker in younger T1D patients, to predict long term changes in kidney function [5–7]. Prior to the development of chronic kidney disease in patients with T1D, it is hypothesized that the effects of type 1 diabetes with hyperglycemia, increased cytokines, advanced glycated end products not only affects the kidneys but also has an effect on bone [8, 9]. Presence of microvascular complications in T1D are associated with increased levels of vascular endothelial growth factor (VEGF) production and action, impairing bone healing [8, 9]. There is also decreased blood vessel formation and reduced bone regeneration [8, 9].

T1D is associated with increased risk of fractures worsened by presence of kidney disease, therefore a major challenge in preventing bone related complication is identifying youth at higher risk. As such, the aim of this study was to evaluate bone health in young adulthood in a cohort of youth with T1D followed longitudinally from adolescence to young adulthood to assess the association of progressive eGFR decline on bone microarchitecture and biomarkers, utilizing second generation HRpQCT imaging and bone biomarkers. Secondary aims were to assess the relationships between HbA_1c_, diabetes duration, BMI, and 25 hydroxy-vitamin D on bone microarchitecture in T1D. The overarching hypothesis is that progressive eGFR decline, in addition to diabetes disease variables, are associated with impairments in bone microarchitecture and biomarkers.

## Methods

### Study population and design

This was a secondary use analysis of longitudinal data of adolescents who participated in the Adolescent Type I Diabetes Cardio-Renal Intervention Trial (AdDIT, EudraCT Number: 2007-001039-72, Trial Registration Number: ISRCTN91419926) in Canada from 2009-2015, and were recruited into a longitudinal, observational study evaluating cardio-renal-bone health in young adults with T1D from 2016 - 2022 at the Hospital for Sick Children in Toronto, Canada [10, 11]. There were pre-specified timelines for data collection in both studies, i.e. AdDIT study baseline, AdDIT midpoint, AdDIT final and cardio-renal-bone study baseline.

This study was approved by the Sick Kids Research Ethics Board. All patients provided informed consent to participate in this study. The study was conducted in accordance with the Declaration of Helsinki.

### Demographic and clinical data

Demographic characteristics including age, sex and ethnicity, along with age of T1D diagnosis, duration of T1D, fracture history (i.e., number and type of fracture), insulin regimen, total daily insulin dose (units/kg/day) and route of administration (pump or injection), history of celiac disease, hypothyroidism, and smoking status were collected from the cardio-renal-bone health study baseline visit. Height, measured using a wall-mounted stadiometer and weight measured by an electronic scale were collected from all study visits.

### Biochemical investigations

HbA_1c_ and creatinine were measured in the Department of Pediatric Laboratory Medicine at SickKids using standard laboratory methods (including an isotope dilution mass spectrometry-traceable assay) and were collected at all four time points. HbA_1c_ was averaged to determine cumulative glycemic exposure.

Serum calcium, phosphate, magnesium, 25 hydroxyvitamin D, bone-specific alkaline phosphatase (Bs-ALP), procollagen-type 1 N-terminal-propeptide (P1NP) and serum cross-linked C-telopeptide (CTX) were measured in the Department of Laboratory Medicine & Pathobiology, Toronto General Hospital, Toronto, Canada using standard laboratory measures. Osteocalcin was measured by Eve Technologies, Calgary, Canada. Osteocalcin was run on multiplex immunoassay and CTX was measured using immunoassay test on Diagnostics e411 analyzer (Roche, Elecsys beta CrossLaps serum assay). Total P1NP and Bs-ALP were run on manual Elisa assay, immunoenzymetric assay with SPECTRA MAX 384 PLUS. Cut-off for Vitamin D sufficiency, insufficiency and deficiency was based on best practice guidelines [12].

### Estimated Glomerular Filtration Rate (eGFR)

eGFR for each study participant was calculated using CKiD U25 creatinine, sex adjusted formula; males = 41.8 x (height /serum creatinine), females = 37.6 x (height /serum creatinine); height in meters, creatinine in mg/dL. This eGFR formula was developed for youth ages 1 – 25, facilitating evaluation of eGFR from adolescence to young adulthood [13]. It has been shown to be the most precise and accurate eGFR equation with the least bias when compared to measured GFR in our cohort [14].

Linear mixed effect regression modeling was used to determine participant-specific eGFR slopes. Participants with eGFR decline $\ge$ 3ml/min/1.73m^2^/year, were classified as progressive eGFR decliners while participants with eGFR $<$ 3 ml/min/1.73m^2^/year were classified as eGFR non-decliners. This eGFR slope cut off was based on prior research studies in patients with diabetes [5–7].

### High Resolution Peripheral Quantitative Computer Tomography (HRpQCT) imaging

In young adulthood, at the final data collection point, images of tibia from the non-dominant ankle and wrist using HRpQCT second generation scanner, (Xtreme CTII, Scanco Medical AG, Brutisellan, Switzerland) was performed on all study participants. The opposite ankle or wrist was scanned if an individual had a history of fracture at the non-dominant side. A trained nuclear medicine technician in performing HRpQCT scans, under supervision of a radiologist performed all scans. Daily and weekly scans were performed as part of quality control. The international guidelines for HRpQCT scanners by Whittier et al., was used to perform the scans [15]. Fixed scanning approach was performed for ankle and wrist measurements and threshold-based approach was used to assess cortical porosity.

### Statistical analysis

Data analysis was conducted using R studio version *1.4.1717*. Normally distributed continuous variables were described as mean ± SD or as median (Q1, Q3), depending on distribution; frequency and proportions were used to describe categorical variables. Linear regression analysis was performed to assess the relationship between eGFR decline and HRpQCT bone parameters, adjusted for age, sex, and body mass index (BMI). To evaluate the relationship between eGFR decline and bone biomarkers, linear regression analysis was performed, adjusting for age, sex and 25 hydroxyvitamin D level. The adjusted covariates were based on prior literature. *P* value less than 0.05 was considered statistically significant for the primary relationship of eGFR decline on HRpQCT bone parameters. *P* value less than or equal to 0.01 was chosen to evaluate secondary outcomes of relationships between demographic and diabetes variables on bone microarchitecture as well as relationship between eGFR decline on bone biomarkers.

## Results

One hundred and three study participants completed HRpQCT scans, four of which had missing data. Total of 99 study participants were evaluated in this study over an average of 7.4 (± 1.0) years. Average age of study participants at time zero was 14.7 (± 1.7) years and 21.3 (± 2.1) years at the final assessment. Median diabetes duration was 13.7 (11.8, 16.0) years with cumulative HbA_1c_ of 8.25% (± 0.95).

Forty four percent (73% male) of study participants were eGFR decliners, compared to 56% (24% male) eGFR non-decliners. The median eGFR slope for the whole cohort was -2.8 (-4.7, -0.7) ml/min/1.73m^2^/year, with the median eGFR slope for eGFR decliner group, -4.9 (-6.2, -4.0) ml/min/1.73m^2^/year and eGFR non-decliner group -0.7 (-2.3, - 0.08) ml/min/1.73m^2^/year.

Among all study participants, 30% reported fracture history with mean number of 0.4 (± 0.8) lifetime fractures. Reported fractures were traumatic fractures, predominantly sport related injuries. There were no significant differences in fracture history or 25 hydroxy-vitamin D level between eGFR decliners and non-decliners (Table 1). Calcium, PTH, phosphate and magnesium were within normal range based on the normative reference ranges of lab assay. Significant differences were found between groups, with eGFR decliner group having a male predominance, younger age at diagnosis and younger age at end of study (Table 1).

**Table 1:** Study participants baseline characteristics based on eGFR decliner and eGFR non-decliner status

Variable	Whole group	eGFR decliners	eGFR non-decliners	*P* value
**N**	99	44 (44.4%)	55 (55.6%)	
**Sex**				
** M: N (%)**	45 (45%)	32 (72.7%)	13 (23.6%)	1.85 x 10^–6^*
** F: N (%)**	54 (55%)	12 (27.3%)	42 (76.4%)	
**Ethnicity**				
** Caucasian**	61 (62%)	26 (59.1%)	35 (63.6%)	0.7
** Non-Caucasian**	38 (38%)	18 (40.9%)	20 (36.4%)	
**Age at end of study (years)** Mean (± SD)	21.3 (± 2.11)	20.4 (± 1.7)	22 (± 2.1)	2.6 x 10^–5^*
**Age of T1D Diagnosis (years)** **Mean (± SD)**	7.5 (± 3.5)	6.5 (± 3.5)	8.3 (± 3.3)	0.006*
**Duration of Diabetes (years)** **Median (Q1,Q3)**	13.71 (11.8, 16.0)	13.2 (11.8, 17.2)	13 (11.8,15.1)	0.8
**eGFR slope ml/min/1.73m**^**2**^**/year** **Median (Q1,Q3)**	-2.8 (-4.7, -0.7)	-4.9 (-6.2, -4.0)	-0.7 (-2.3, -0.08)	< 2.2 x 10^–16^*
**Baseline Hyperfiltration (eGFR ≥ 135 ml/min/1.73m**^**2**^**) N (%)**	7 (7%)	2 (4.5%)	5 (9%)	0.5
**Height (cm)** **Mean (± SD)**	169.1 (± 14.2)	171.7 (± 19.0)	167.1 (± 8.2)	0.9
**BMI (kg/m**^**2**^**)** **Mean (± SD)**	25.7 (± 5.5)	25.05 (± 5.2)	26.2 (± 5.7)	0.1
**Final HbA**_**1c**_**(%)** **Mean (± SD)**	8.06 (± 1.3)	8.0 (± 1.0)	8.1 (± 1.5)	0.2
**Cumulative HbA**_**1c**_**(%)** **Mean (± SD)**	8.25 (± 0.95)	8.2 (± 0.8)	8.3 (± 1.1)	0.3
**25 hydroxy vitamin D (nmol/L)** **Median (Q1,Q3)**	54.7 (35.8, 75.2)	57.2 (34.3, 77.2)	53.9 (38.7, 73.4)	0.7
**Vitamin D Status N (%)**				
** Deficient (< 50 nmol/L)**	44 (44%)	20 (45.5%)	24 (43.6%)	0.9
** Intermediate(50–74.9 nmol/L)**	29 (29%)	12 (27.3%)	17 (30.9%)	
** Sufficient (≥75 nmol/L)**	26 (26%)	12 (27.3%)	14 (25.5%)	
**PTH (pmol/L)** **Mean (± SD)**	6.47 (± 6.06)	5.4 (± 1.8)	7.3 (± 7.9)	0.06
**Calcium (mmol/L)** **Mean (± SD)**	2.4 (± 0.09)	2.4 (± 0.09)	2.4 (± 0.09)	1.0
**Phosphate (mmol/L)** **Mean (± SD)**	1.4 (± 0.2)	1.4 (± 0.2)	1.4 (± 0.2)	0.5
**Magnesium (mmol/L)** **Mean (± SD)**	0.8 (± 0.06)	0.8 (± 0.05)	0.7 (± 0.06)	0.9
**Fracture History**				
** Yes – N (%)**	30 (30%)	12 (27%)	18 (33%)	0.8
** No – N (%)**	49 (49%)	22 (50%)	27 (49%)	
** Unknown – N (%)**	20 (20%)	10 (23%)	10 (18%)	
**Number of Fractures**				
**Mean (± SD)**	0.43 (± 0.8)	0.43 (± 0.8)	0.4 (± 0.8)	0.5
**Range**	0-4	0 - 3	0-4	
** Insulin Regimen**				
** Injection N (%)**	37 (37%)	16 (36.4%)	21 (38.2%)	1
** Pump N (%)**	62 (63%)	28 (63.6%)	34 (61.8%)	
**TDD (units/kg/day)** **Median (Q1,Q3)**	0.8 (0.6, 0.9)	0.8 (0.7,0.9)	0.8 (0.6, 1.0)	0.6
**Celiac Disease N (%)**	5 (5%)	4 (9.1%)	1 (1.8%)	0.2
**Hypothyroidism N (%)**	2 (2%)	1 (2.3%)	1 (1.8%	1
**Smoking N (%)**	4 (4%)	3 (6.8%)	1 (1.8%)	0.3

Data are shown as mean ± SD, median (Q1,Q3), or in numbers and percentages as appropriate. Between group differences were assessed using unpaired t-test, Mann-Whitney U test and fisher’s exact test as appropriate

^*^Significant difference between both groups at *p* value of 0.05

### Primary outcomes: HRpQCT

In the adjusted linear regression analysis, being in the eGFR decliner group was associated with 5% higher tibia cortical porosity (*p* = 0.035). There were no other statistically significant associations between eGFR decline and other HRpQCT bone parameters (Table 2).

**Table 2:** Relationship between HRpQCT bone parameters at tibia and radius and eGFR decliner status

HRpQCT Parameter	Tibia decliner group Mean (± SD)	Tibia non-decliner group Mean (± SD)	Adjusted *p* value	Radius decliner group Mean (± SD)	Radius non-decliner Mean (± SD)	Adjusted *p* value
**N**	44	55		44	55	
**Total vBMD (mgHA/cm**^**3**^**)**	332.9 (± 50.5)	335.6 (± 60.75)	0.9	391.1 (± 65.58)	396.9 (± 79.53)	0.15
**Total Area (mm**^**2**^**)**	762.7 (± 171.2)	671.1 (± 137.39)	0.07	256.7 (± 52.9)	228.8 (± 52.67)	0.4
**Ct BMD (mgHA/cm**^**3**^**)**	925.8 (± 56.88)	953.1 (± 48.42)	0.16	931.7 (± 54.22)	964.0 (± 84.62)	0.96
**Ct Area (mm**^**2**^**)**	141.4 (± 27.8)	133.4 (± 27.0)	0.8	70.3 (± 14.5)	71.5 (± 14.2)	0.2
**CtTh (mm)**	1.54 (± 0.32)	1.56 (± 0.336)	0.67	1.35 (± 0.26)	1.30 (± 0.25)	0.477
**Ct.Pm (mm)**	106.8 (± 11.46)	100.07 (± 10.2)	0.99	65.60 (± 7.66)	61.24 (± 7.39)	0.14
**Ct.Po (%)**	1.5 (± 1.0)	1.55 (± 1.0)	0.11	0.47 (± 0.4)	0.47 (± 0.5)	0.3
**Ct.Po.Dm (mm)**	0.22 (± 0.03)	0.21 (± 0.04)	0.035*	0.15 (± 0.027)	0.15 (± 0.03)	0.88
**Tb BMD (mgHA/cm**^**3**^**)**	193.6 (± 29.0)	179.3 (± 37.22)	0.5	167.7 (± 38.99)	148.7 (± 47.98)	0.33
**Tb Area (mm**^**2**^**)**	630.6 (± 163.8)	544.3 (± 131.0)	0.95	176.6 (± 50.0)	171.0 (± 50.1)	1.0
**Tb.N (1/mm)**	1.44 (± 0.21)	1.38 (± 0.197)	0.41	1.48 (± 0.19)	1.4 (± 0.24)	0.6
**Tb.Sp (mm)**	0.65 (± 0.1)	0.69 (± 0.1)	0.60	0.63 (± 0.098)	0.69 (± 0.15)	0.98
**Tb.Th (mm)**	0.26 (± 0.023)	0.25 (± 0.023)	0.89	0.23 (± 0.02)	0.22 (± 0.02)	0.5

*vBMD* volumetric bone mineral density, *Ct*cortical, *Th* thickness, *Tb* trabecular, *Sp* separation, *N* Number, *Ct*. *Pm* cortical perimeter, *Ct*. *Po* cortical porosity, *Ct*.*Po*.*Dm* cortical porosity diameter

^*^significance at 0.05 in linear regression model adjusted for covariates age, sex, and BMI

### Secondary outcomes

**Fig. 1:**
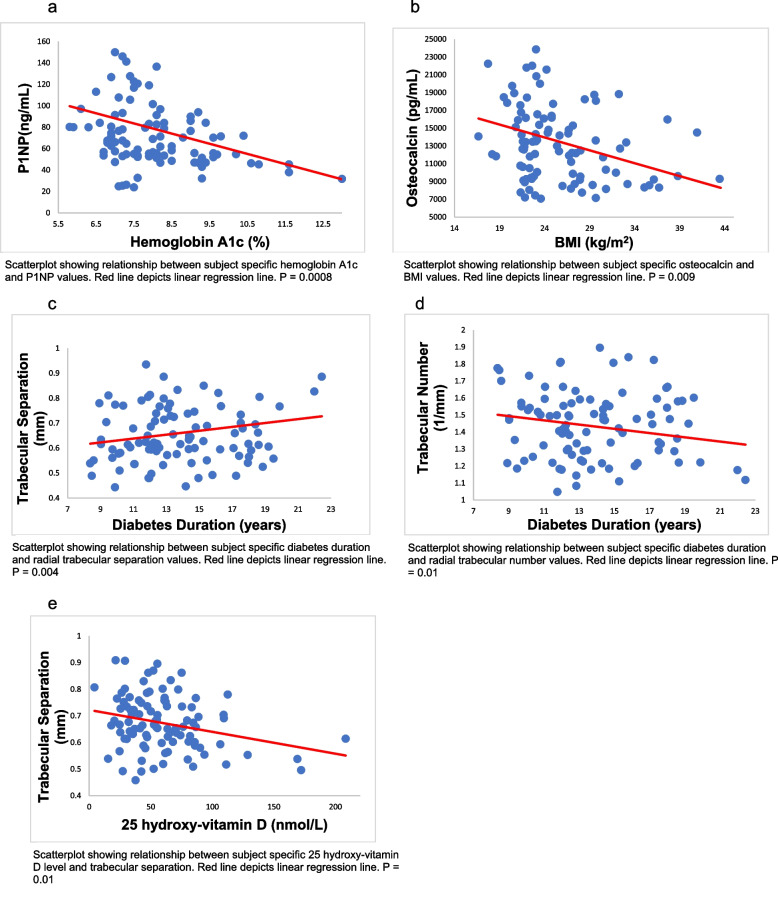
Relationships between diabetes and demographic variables and markers of bone health

In the adjusted model, there were no statistically significant associations between progressive eGFR decline and bone biomarkers (Table 3). Higher HbA_1c_ was associated with lower P1NP (*p* = 0.0008) (Fig. 1a). Increasing BMI was associated with lower osteocalcin (*p* = 0.009), (Fig. 1b). Increasing diabetes duration was associated with increasing radius trabecular separation (*p* = 0.004) and decreasing radius trabecular number (*p* = 0.01), (Fig. 1 c, d). Lower 25 hydroxyvitamin D concentration was associated with higher tibia trabecular separation (*p* = 0.01), (Fig. 1e). The relationship between HbA_1c,_ diabetes duration, BMI and vitamin D level and bone microarchitecture at the tibia and radius are outlined in Tables 4 and 5.

**Table 3:** Relationship between bone biomarkers and eGFR decliner status

Variable	Whole cohort mean (± SD)	eGFR decliner mean (± SD)	eGFR non decliner mean (± SD)	Adjusted *p* value
**Osteocalcin (pg/mL)**	13448 (± 4882.1)	15443 (± 5461.8)	11889 (± 3737.3)	0.3
**CTX (ng/mL)**	0.57 (± 0.3)	0.65 (± 0.32)	0.51 (± 0.28)	0.2
**P1NP (ng/mL)**	78.3 (± 44)	93.95 (± 56.4)	65.9 (± 25.3)	0.7
**Bs-ALP (** ${\varvec{u}}$**g/L)**	20.6 (± 10)	23.9 (± 12.8)	17.9 (± 6.2)	0.5

*CTX* c-terminal telopeptide, *P1NP* procollagen 1 intact n-terminal pro-peptide and *Bs-ALP* bone specific alkaline phosphatase

Linear regression analysis with adjustment for covariates age, sex and 25 hydroxyvitamin D was performed

**Table 4:** Relationship between tibia HRpQCT parameters and demographic and diabetes variables

HRpQCT parameters	Hemoglobin A1C			Diabetes duration			BMI			Vitamin D level		
	*R*^2^	*β*	p	*R*^2^	*β*	p	*R*^2^	*β*	p	*R*^2^	*β*	p
Tt.BMD (mgHA/cm^3^)	0.1	7.4	0.08	0.08	0.2	0.9	0.09	0.7	0.4	0.08	0.02	0.9
Tt. Ar (mm^2^)	0.3	5.0	0.7	0.3	-8.4	0.06	0.3	3.5	0.06	0.2	-0.2	0.7
Ct.BMD (mgHA/cm^3^)	0.3	1.7	0.6	0.3	1.8	0.2	0.3	0.4	0.6	0.3	-0.2	0.2
Ct.Ar (mm^2^)	0.35	3.2	0.07	0.3	-0.8	0.3	0.3	0.9	0.003*	0.3	-0.08	0.3
Ct.Th (mm)	0.1	0.04	0.07	0.1	1x10^-4^	1.0	0.1	0.006	0.2	0.1	-8x10^-4^	0.4
Ct.Pm (mm)	0.3	0.1	0.9	0.3	-0.6	0.05	0.3	0.3	0.03	0.3	-0.01	0.7
Ct.Po (%)	0.1	7x10^-4^	0.2	0.1	-2x10^-4^	0.4	0.1	-5x10^-6^	1.0	0.1	2x10^-5^	0.4
Ct.Po.Dm (mm)	0.1	4x10^-3^	0.1	0.1	-4x10^-5^	1.0	0.1	1x10^-4^	0.8	0.1	-4x10^-5^	0.7
Tb.BMD (mgHA/cm^3^)	0.3	4.4	0.05	0.3	-1.5	0.1	0.3	0.6	0.2	0.3	0.1	0.2
Tb.Ar (mm^2^)	0.2	2.0	0.9	0.2	-7.7	0.08	0.2	2.9	0.1	0.2	-0.08	0.8
Tb.N (1/mm)	0.08	0.008	0.6	0.1	-0.01	0.06	0.09	0.003	0.3	0.1	0.001	0.02
Tb.Sp (mm)	0.08	-0.008	0.3	0.09	0.005	0.1	0.08	-0.001	0.4	0.1	-8x10^-4^	0.01*
Tb.Th (mm)	0.2	0.002	0.33	0.2	3x10^-4^	0.6	0.2	3x10^-4^	0.3	0.3	-9x10^-5^	0.2

*vBMD* volumetric bone mineral density, *Ct*cortical, *Th* thickness, *Tb* trabecular, *Sp* separation, *N* Number, *Ct*. *Pm* cortical perimeter, *Ct*. *Po* cortical porosity, *Ct*.*Po*.*Dm* cortical porosity diameter

^*^Significance at *p* $\le$0.01. linear regression analysis with adjustment of covariates of relationship between HbA1C, diabetes duration, body mass index, 25 hydroxy-vitamin D level, and tibia HRpQCT parameters. Adjusted *R*^2^, beta coefficient and *p* values are reported

**Table 5:** Relationship between radius HRpQCT parameters and demographic and diabetes variables

HRpQCT parameters	Hemoglobin A1C			Diabetes duration			BMI				Vitamin D level		
	*R*^2^	*β*	p	*R*^2^	*β*	p	*R*^2^	*β*		p	*R*^2^	*β*	p
Tt BMD (mgHA/cm^3^)	0.08	-1.2	0.8	0.09	1.3	0.6	0.1		0.2	0.8	0.09	-0.02	0.9
Tt. Ar (mm^2^)	0.1	3.0	0.5	0.2	-2.9	0.08	0.1		1.5	0.03	0.1	0.1	0.5
Ct BMD (mgHA/cm^3^)	0.1	-1.8	0.7	0.1	4.0	0.08	0.1		0.5	0.6	0.1	-0.1	0.5
Ct. Ar (mm^2^)	-0.03	0.5	0.7	-0.03	-0.3	0.6	-0.02		-0.1	0.6	-0.03	0.03	0.6
Ct.Th (mm)	0.07	-0.01	0.6	0.08	0.01	0.2	0.07		0.003	0.4	0.06	0.0001	0.9
Ct Pm (mm)	0.1	0.8	0.1	0.1	-0.2	0.4	0.1		0.1	0.1	0.1	0.02	0.5
Ct.Po (%)	-0.01	-0.6	1.0	-0.003	0.3	0.2	-0.01		-0.03	0.8	-0.02	-0.02	0.4
Ct.Po.Dm (mm)	-0.01	-1.5	0.3	-0.003	0.8	0.2	-0.01		-0.07	0.8	-0.02	-0.05	0.4
Tb BMD (mgHA/cm^3^)	0.4	2.6	0.4	0.4	-2.4	0.03	0.4		0.05	0.9	0.4	0.2	0.2
Tb.Ar (mm^2^)	0.02	-1.7	0.7	0.02	0.8	0.6	0.03		-1.3	0.05	0.02	0.1	0.4
Tb. N (1/mm)	0.1	4x10^-3^	0.8	0.2	-0.02	0.01*	0.1		1x10^-5^	1.0	0.2	1x10^-3^	0.03
Tb. Sp (mm)	0.2	-0.003	0.7	0.2	0.01	0.004*	0.2		-5x10^-4^	0.8	0.2	-8x10^-4^	0.04
Tb. Th (mm)	0.4	0.002	0.3	0.4	1x10^-5^	1.0	0.4		-8x10^-5^	0.7	0.4	5x10^-5^	0.4

*vBMD* volumetric bone mineral density, *Ct*cortical, *Th* thickness, *Tb* trabecular, *Sp* separation, *N* Number, *Ct*. *Pm* cortical perimeter, *Ct*. *Po* cortical porosity, *Ct*.*Po*.*Dm* cortical porosity diameter

Significance at *p* $\le$0.01. linear regression analysis with adjustment of covariates of relationship between HbA1C, diabetes duration, body mass index, 25 hydroxy-vitamin D level, and radius HRpQCT parameters. Adjusted *R*^2^, beta coefficient and *p* values are reported

## Discussion

The primary objective of this study was to assess the effects of progressive eGFR decline, an early marker of microvascular kidney injury in T1D on bone microarchitecture [5–7]. A statistically significant change was found with 5% higher cortical porosity diameter in young adults with T1D and progressive eGFR decline compared to participants with a lack of eGFR decline. This is a novel finding, not previously reported. This is likely due to the study’s use of second generation HRpQCT, which provides better resolution in contrast to previous reported studies using first generation HRpQCT. Previous studies have reported changes in cortical porosity; a recent study of adolescents with T1D showed a 21.5% increase in cortical porosity in participants with T1D compared to healthy controls [4]. It is proposed that a change in cortical porosity diameter would occur prior to cortical porosity.

Although a statistically significant difference was found in cortical porosity diameter, there were no other significant differences among the other HRpQCT parameters assessing bone microarchitecture or with bone biomarkers. The limited findings of microarchitectural changes in this study may be due to the young adult age of the subjects which is when peak bone mass is achieved. Additionally, shorter diabetes duration and limited glycemic exposure could limit the extent of the effects of advanced glycated end products, glycemia and oxidative stress on microvasculature and bone re-modeling [8].

T1D has been shown to be associated with a low bone turnover state and reduced BMD [16]. Presence of kidney disease in T1D has features of exocrine insufficiency, and a degree of malabsorption that can lead to lowered BMD and mineralization abnormalities [16]. Hyperglycemia may be associated with increased loss of calcium in urine, however due to other regulatory systems, most notably parathyroid hormone, serum calcium is usually maintained [17]. Lower levels of 25 hydroxyvitamin D and higher levels of trabecular separation was found in this study. A systematic review and meta-analysis showed a 2.6% increase in total body bone mineral content and 1.7% increase in lumbar spine bone mineral density from baseline in children with low vitamin D who were supplemented [18].

In this study, higher HbA_1c_ was associated with lower levels of P1NP, a marker of bone formation. A recent study found lower P1NP with higher HbA_1c_ in adolescents with T1D [4]. Secondary analysis of the Diabetes Control and Complications Trial (DCCT)/ Epidemiology of Diabetes Interventions and Complications (EDIC) study found worse glycemic control to be associated with lowered bone formation [19]. This finding is likely due to the negative effects of high glucose on osteoblast response to mechanical loading and differentiation of mesenchymal stem cells [20, 21].

In a recent adolescent study with sub-analysis of patients with T1D duration of 2 years or longer, an association was found with lower trabecular number and increased trabecular separation [22]. Similar microarchitectural changes were observed in this study with longer diabetes duration having an association with higher trabecular separation and lower trabecular number.

T1D was previously associated with a lean phenotype, however there are recent reports of increasing rates of overweight and obesity in T1D [23]. The average BMI of this cohort was 25.7kg/m^2^, which is in the overweight category in BMI classification [24]. Increasing BMI was associated with lower levels of osteocalcin, a marker of bone formation. There is conflicting evidence regarding BMI and its effect on bone health [25]. There is evidence of potential role of high BMI as a protective factor to bone loss with it increasing bone mineral density due to higher levels of 17 ß-estradiol, higher mechanical load and decreasing bone turnover [25]. However, there is concern of potential microarchitectural changes that might be resulting in increased fracture rates observed with obesity [26].

While this study showed a small but statistically significant difference between cortical porosity diameter and progressive eGFR decline; it is noted that there were minimal differences observed in the other HRpQCT parameters and bone biomarkers in the adjusted linear regression analysis. Further longitudinal HRpQCT studies are needed to better understand the long-term impact of progressive eGFR decline on bone microarchitecture and biomarkers and fracture risk.

There are a few study limitations. This study was a secondary analysis of previously collected data, and the initial goal of the original research study was not specifically to address this study’s hypothesis. This cohort had overall good metabolic control and high technology, pump use which may correspond to lower elevations in glycemic exposure and reduced glucotoxicity effect on bone microarchitecture and biomarkers. HRpQCT provides extensive information with multiple parameters assessed at the tibia and radius, therefore a well-developed statistical plan was established a priori to the analysis, in order to limit potential type 1 error. There was a lack of study participants with substantial reduced kidney function. eGFR decline was chosen as a surrogate marker of early kidney disease in T1D for this study based on previous longitudinal studies, however another surrogate marker or addition of multiple markers could have provided better evaluation of early signs of microvascular changes in the kidney [5–7, 27]. The study population was primarily Caucasian, 62%, therefore lack of ability to evaluate ethnicity related factors.

Strengths of this study include addressing gaps in literature and specifically, the evaluation of the impact of early signs of kidney microvascular disease in adolescents with T1D on bone microarchitecture in young adulthood. A second generation HRpQCT scanner was used which has better resolution, providing improved evaluation of bone micro-architecture. Additionally, this study utilized the updated CKiD U25 formulae which allows evaluation of eGFR from childhood to young adulthood. This study was a secondary analysis of a well phenotype longitudinally evaluated cohort, providing a wealth of information.

## Conclusion

In summary, while a single outcome with 5% increased cortical porosity diameter was observed to be statistically significant in the eGFR decliner group compared to those with stable eGFR, overall measures of HR-pQCT and bone biomarkers were similar between both groups and were not statistically significant. These data suggest skeletal impairments are limited with youth with T1D near peak bone mass with different eGFR trajectories. Significant relationships were found with markers of increased skeletal fragility; increased diabetes duration, higher HbA_1c_, lower 25 hydroxyvitamin D and higher BMI. While further studies with longitudinal HR-pQCT evaluation are needed to determine the long-term effects of progressive eGFR decline on bone microarchitecture and fracture risk, these results emphasize the importance of optimal glycemic, blood pressure control, healthy lifestyle with weight bearing physical activity, along with appropriate dietary calcium and vitamin D intake in patients with T1D.

## Data Availability

All data generated or analyzed during this study are available from the corresponding author on reasonable request.

## Abbreviations

Bs-ALP: Bone specific alkaline phosphatase
Ct: Cortical
Ct.Pm: Cortical perimeter
Ct.Po: Cortical porosity
Ct.Po.Dm: Cortical porosity diameter
CTX: C-terminal telopeptidee
GFR: Estimated glomerular filtration rate
HbA_1c_: Glycated hemoglobin
HRpQCT: High resolution peripheral quantitative computed tomography
N: Number
P1NP: Procollagen 1 intact n-terminal pro-peptide
Sp: Separation
Tb: Trabecular
T1D: Type 1 diabetes
Th: Thickness
vBMD: Volumetric bone mineral density
